# Automaticity of the Pulmonary Vein Myocardium and the Effect of Class I Antiarrhythmic Drugs

**DOI:** 10.3390/ijms252212367

**Published:** 2024-11-18

**Authors:** Iyuki Namekata, Maika Seki, Taro Saito, Ryosuke Odaka, Shogo Hamaguchi, Hikaru Tanaka

**Affiliations:** Department of Pharmacology, Faculty of Pharmaceutical Sciences, Toho University, 2-2-1 Miyama Funabashi, Chiba 274-8510, Japan; iyuki@phar.toho-u.ac.jp (I.N.); 3024002s@st.toho-u.ac.jp (M.S.); 3022005s@st.toho-u.jp (T.S.); 3021002o@st.toho-u.jp (R.O.); shogo.hamaguchi@phar.toho-u.ac.jp (S.H.)

**Keywords:** pulmonary vein myocardium, automaticity, intracellular Ca^2+^, late I_Na_, class I antiarrhythmics, atrial fibrillation

## Abstract

The pulmonary vein wall contains a myocardial layer whose ectopic automaticity is the major cause of atrial fibrillation. This review summarizes the results obtained in isolated pulmonary vein myocardium from small experimental animals, focusing on the studies with the guinea pig. The diversity in the action potential waveform reflects the difference in the repolarizing potassium channel currents involved. The diastolic depolarization, the trigger of automatic action potentials, is caused by multiple membrane currents, including the Na^+^-Ca^2+^ exchanger current and late I_Na_. The action potential waveform and automaticity are affected differentially by α- and β-adrenoceptor stimulation. Class I antiarrhythmic drugs block the propagation of ectopic electrical activity of the pulmonary vein myocardium through blockade of the peak I_Na_. Some of the class I antiarrhythmic drugs block the late I_Na_ and inhibit pulmonary vein automaticity. The negative inotropic and chronotropic effects of class I antiarrhythmic drugs could be largely attributed to their blocking effect on the Ca^2+^ channel rather than the Na^+^ channel. Such a comprehensive understanding of pulmonary vein automaticity and class I antiarrhythmic drugs would lead to an improvement in pharmacotherapy and the development of novel therapeutic agents for atrial fibrillation.

## 1. Introduction

The pulmonary vein, the blood vessel connecting the lung and the left atrium, shows spontaneous pulsation independent of the cardiac cavity; this was reported as early as the 19th century [1]. The pulmonary vein contains a myocardial layer connected to the atrial myocardium which can generate spontaneous or triggered action potentials [2,3]. The generation of the action potential and its waveform show diverse patterns and are affected by neurohumoral substances. Since the clinical report that paroxysmal atrial fibrillation is initiated by ectopic action potential generated in the pulmonary veins [4,5], the automatic activity of the pulmonary vein myocardium has received attention as a target for the pharmacological treatment of atrial fibrillation. Class I antiarrhythmic (antidysrhythmic) drugs, which are one of the first-line drugs for atrial fibrillation, affect the electrical activity of the pulmonary vein myocardium differentially. The physiological and pharmacological properties of the pulmonary vein myocardium have been studied in various experimental animals [6]. In this review, we will summarize the information obtained in the pulmonary vein myocardium of small experimental animals focusing on our own studies with the guinea pig.

## 2. Permissive Role of Reduced Repolarizing Current Density

**Figure 1:**
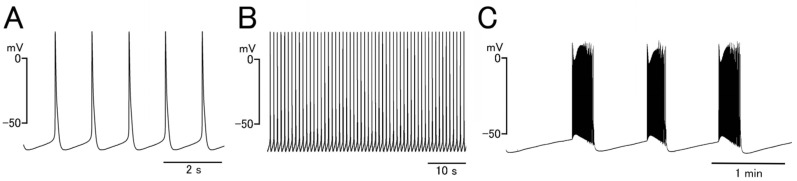
Automatic electrical activity of the pulmonary vein myocardium. (**A**) Spontaneous action potential recording from the guinea pig showing a diastolic depolarization. (**B**) Continuous firing in the guinea pig. (**C**) Repetitive burst-type firing in the rat elicited by the application of 1 μM noradrenaline.

The cardiomyocytes from the pulmonary vein myocardium are roughly similar to those from the atria in size and morphology. In the case of the guinea pig, they form a myocardial layer in between the smooth muscle layer and the adventitia [7]. The key factor for the manifestation of automaticity in the pulmonary vein myocardium is the weak repolarization power of the cardiomyocyte to maintain its resting membrane potential. Microelectrode experiments with isolated guinea pulmonary vein tissue preparations revealed the presence of spontaneous electrical activity in about 30% of the preparations (Figure 1A); such activity was not observed in preparations from the atria or ventricle [2,3,8,9]. The remaining quiescent preparations had a resting membrane potential that was less negative than the atrial and ventricular myocardium. When they were driven by field electrical stimulation, they generated action potentials accompanied by a diastolic depolarization. A less negative resting membrane potential and automatic activity have been reported in the pulmonary vein myocardium of other experimental animal species, including the rabbit [10], dog [11], rat [12], and mouse [13], but not in the atrial myocardium. This implies that the pulmonary vein cardiomyocytes have less stability of membrane potential than the atrial and ventricular cardiomyocytes.

The potassium currents responsible for the stability of the resting membrane potential are the inwardly rectifying potassium current I_K1_ and the acetylcholine-activated potassium current I_KACh_. These currents both have an inwardly rectifying current–voltage relationship. I_K1_ is the main current for the maintenance of the resting membrane potential in the working myocardium. I_KACh_ is induced in atrial cardiomyocytes by the stimulation of muscarinic acetylcholine and adenosine receptors, but, in some cases, flows in the absence of agonists, as in the case of the guinea pig pulmonary vein cardiomyocytes [9]. An agonist-independent I_KACh_ was also reported in the guinea pig atrial [14] and canine atrial [15] myocardium. The total inwardly rectifying potassium current of guinea pig pulmonary vein cardiomyocytes had a current–voltage relationship similar to, but a current density significantly lower than, that of the atrial myocytes [9]. The lower density of the inwardly rectifying potassium current, resulting in less negative membrane potential, was also reported in the rabbit [10] and dog [11]. A positive shift in the resting membrane potential by tertiapin, which blocks I_KACh_, was reported in the pulmonary vein myocardium of the guinea pig [9] and rat [15], but not in the atrial. The less inwardly rectifying potassium current reserve probably makes the membrane potential of the pulmonary vein myocardium more sensitive to pharmacological agents than the atrial and ventricular myocardium.

In the quiescent pulmonary vein myocardium of the guinea pig, the application of tertiapin or a high-frequency field stimulation induced automatic electrical activity [9], a phenomenon not observed in normal atrial and ventricular myocardia [16]. Such automatic electrical activity was completely inhibited by carbachol, which induces I_KACh_ and increases the total inwardly rectifying potassium current density. These results indicate that the main factor that allows the generation of spontaneous electrical activity in the pulmonary vein myocardium is the lower density of the total inwardly-rectifying potassium current. In the case of the stimulation-evoked action potential in the rat pulmonary vein myocardium, tertiapin caused depolarization of the resting membrane potential and inhibited the generation of action potentials [15]. Thus, the effect of the resting membrane potential on action potential generation may not be simple.

The automaticity of the isolated pulmonary vein can be affected by the pathological state of the animal. The abdominal aorto-venocaval shunt rat is characterized by volume overload to the heart and is considered to be in a state of high-output heart failure. The duration of experimental atrial fibrillation induced by burst pacing was significantly longer than that of Sham-operated rats, which was accompanied by changes in the atrial expression levels of potassium channels [17]. At 12 weeks after the shunt operation, a reduction in the atrial mRNA expression of Kv1.5, Kv4.2, and Kv4.3, which encodes the I_Kur_ and the transient outward current (I_to_), was observed; the changes were larger in the right atria than the left. Kv4.2 and Kv4.3, which encode the I_to_, were also decreased in the right atria. The expression of Nav1.5, which encodes the Na^+^ channel, was not decreased. These are consistent with the maintained maximum rate of rise of the action potential (phase 0) and the slight prolongation of the action potential duration at 90% repolarization (APD_90_). Thus, the function of the Na^+^ channel is maintained in this model. The pulmonary vein preparations from the aorto-venocaval shunt rat had a less negative resting membrane potential, a longer action potential duration, and a tendency to generate spontaneous electrical activity than those from the normal rats [18]. It is probable that a decrease in the repolarizing power of the pulmonary vein myocardium underlies its tendency to develop atrial fibrillation.

The chronic atrioventricular (AV) block dog, another model of volume overload, is considered to be in a state of low-output heart failure [19]. Paroxysmal atrial fibrillation was observed immediately after the AV block operation, and the duration of the burst-pacing-induced atrial fibrillation was progressively increased. After four weeks, these dogs could be used as an early-stage atrial fibrillation model [20]. Pilsicainide (1 mg/kg), which significantly prolonged the inter-atrial conduction time, suppressed the burst pacing-induced atrial fibrillation by 58%, which suggests that blockade of the Na^+^ channel is effective for early-stage atrial fibrillation. Further application of tachypacing to the atrium of AV block dogs for more than 6 weeks resulted in the completion of the electrical and structural remodeling of the atria which resembled the pathology of the persistent atrial fibrillation [21,22]. The persistent atrial fibrillation of this model could not be suppressed by apridine, suggesting that simple blockade of Na^+^ channels is ineffective against persistent atrial fibrillation. Pilsicainide (3 mg/kg) terminated the atrial fibrillation in only 25% of the animals examined. In this experiment, pilsicainide significantly prolonged the inter-atrial conduction time, which indicates that the Na^+^ channel itself was effectively blocked in the remodeled myocardium. It was reported that the effect of the Na^+^ channel blockade is altered in the remodeled myocardium [23]. In contrast, the I_KACh_ blocker AVE0118 effectively suppressed the persistent atrial fibrillation, which indicates that additional mechanisms of action, such as the blockade of K^+^ channels, are necessary at this stage [21]. The action potential of the pulmonary vein myocardium became significantly shorter after chronic AV block, which was caused by an increase in the expression of intermediate Ca^2+^-activated K^+^ channels [24]. In these two models of cardiac volume overload, changes in the concentration of various circulating bioactive substances were reported. Thus, both mechanical and humoral factors have a great influence on the automaticity of the pulmonary vein myocardium. Thus, the pathological changes in K^+^ currents and their relation to the automaticity of the pulmonary vein myocardium appear to be complex.

## 3. Species Difference in Action Potential Waveform

The firing pattern and waveform of the pulmonary vein action potentials were different among experimental animal species, and, in most cases, a continuous firing of action potentials was observed [13,25]. In the guinea pig pulmonary vein myocardium, repetitive firing was observed in about half of the tissue preparations and the average frequency of continuous firing was about 1 Hz (Figure 1A) [8]. Almost all of the quiescent preparations showed automatic activity after noradrenaline treatment. The pulmonary vein myocardium had a less negative resting membrane potential and smaller maximum upstroke velocity and amplitude of the action potential than the atrial myocardium [3,8]. Similar results have been reported in the canine pulmonary vein myocardium [11]. As the expression level of the voltage-dependent Na^+^ current was not different, the smaller maximum upstroke velocity in the pulmonary vein could be explained by the less negative resting membrane potential.

In the case of the rat and mouse pulmonary vein [13,26], automatic activities appeared as repetitive bursts, which was different from the continuous firing observed in the guinea pig and canine pulmonary veins. In the case of the rat pulmonary vein, the incidence of spontaneous electrical firing was very low under basal conditions but was increased in the presence of adrenergic stimuli; the waveform was exclusively the burst type (Figure 1C). In the case of the mouse pulmonary vein myocardium, spontaneous activity of both the continuous type and the burst type was observed. The burst-type waveform and the gradual negative shift of the maximum diastolic potential during the burst imply a periodic change in the balance between depolarizing and repolarizing power. Accumulation of certain ion channel states and/or changes in intracellular ion concentrations during the burst may affect the membrane currents, and eventually inhibit the firing of action potentials.

**Figure 2:**
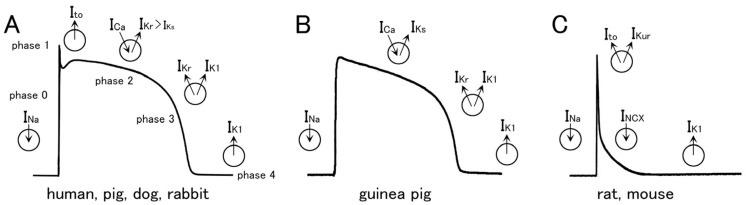
A simplified schematic diagram of the action potential configuration and the underlying ionic currents in different species. (**A**) Human, pig, dog, and rabbit. (**B**) Guinea pig. (**C**) Rat and mouse.

The species difference in the action potential waveform, which has been most extensively studied in the working myocardium, could largely be attributed to a difference in the repolarizing potassium channel currents (Figure 2; [27,28,29,30,31,32,33]). In humans, pigs, dogs, and rabbits (Figure 2A), the resting membrane potential (phase 4) is maintained by the I_K1_. The rapid depolarization (phase 0) is caused by the Na^+^ channel current followed by a rapid and partial repolarization caused by the transient outward current (Ito) to form a notch (phase 1). The plateau phase (phase 2) is caused by a balance between the inward flow of the L-type Ca^2+^ channel current (I_CaL_) and the gradually activating repolarizing K^+^ currents. The delayed rectifying K^+^ channel current (I_K_) is responsible for the early repolarization (phase 3). This current is composed of the rapidly activating component (I_Kr_) and the slowly activating component (I_Ks_); in most animals, the former component plays a larger role. I_K1_ also contributes to the later phase of repolarization. In the case of the guinea pig (Figure 2B), the I_Ks_ play a dominant role in repolarization and the role of I_Kr_ and I_to_ is small or lacking. Thus, the guinea pig action potential lacks the notch (phase 1). In the case of the rat and mouse (Figure 2C), repolarization is mainly caused by Ito and I_Kur_, resulting in an extremely short action potential duration at depolarized membrane potentials (phase 3). The rat and mouse have characteristic electrophysiological, Ca^2+^-handling, and metabolic properties, which appear to support the high heart rate in these species [34,35,36,37,38]. In the rat and mouse ventricular myocardium under artificial stimulation at low frequencies, a relatively slow repolarization at negative membrane potentials is observed, which is sometimes called the late plateau phase [39]. This phase is caused by the extrusion of intracellular Ca^2+^ by the Na^+^-Ca^2+^ exchanger; the lowered repolarizing power at this phase due to the rapid inactivation of Ito probably permits the manifestation of the depolarizing effect of the Na^+^-Ca^2+^ exchanger current. The role of I_Kr_ is very small in the rat and mouse.

The species difference in the function of the I_Na_ and I_CaL_, as well as their influence on the action potential properties, appear to be small compared with the K^+^ currents [32,40]; the fading of the I_CaL_ during the action potential is largely determined by the action potential configuration [28]. Pilsicainide, which selectively blocks the Na^+^ channel, is effective in various animal species to block the I_Na_, reduce the maximum rate of rise of the action potential, and decrease the conduction velocity [41,42,43,44,45,46]. Thus, class I antiarrhythmic drugs can be expected to reduce the myocardial conduction velocity and be effective against atrial fibrillation. The class I antiarrhythmic drug flecainide was reported to reduce the maximum rate of rise of the action potential similarly in atrial myocardia from the human, rabbit, dog, and guinea pig [47]. The sensitivity to flecainide was slightly higher in humans than in animal species; the effect of 2.25 μM of the drug in humans was comparable to that of 4.5 μM in animal species. Also, the effect was stronger under higher firing frequencies; the rate dependency was greatest in the human myocardium and the least in the guinea pig.

The pulmonary vein myocardium has a low repolarization power to maintain the resting membrane potential, as mentioned above. This allows the otherwise masked depolarizing effects of various membrane currents, including the Na^+^-Ca^2+^ exchanger current and late Na^+^ current mentioned below, and the hyperpolarization-activated current (I_f_). The properties of the diastolic depolarization of the pulmonary vein myocardium, as well as the pattern of action potential firing (continuous vs. burst), appear to be caused by differences in the repolarizing membrane currents.

## 4. Involvement of Intracellular Ca^2+^-Dependent Mechanisms

**Figure 3:**
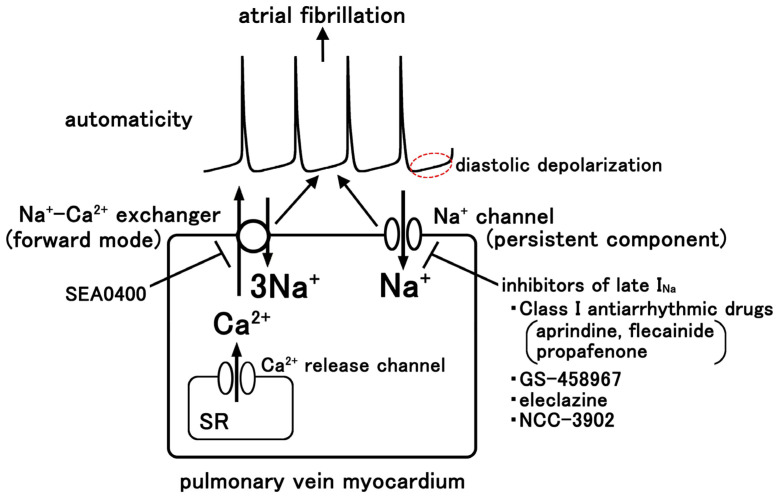
A scheme for the automatic electrical activity of the pulmonary vein myocardium. The forward-mode Na^+^-Ca^2+^ exchanger and the persistent component of the Na^+^ channel current (late I_Na_) depolarize the cell membrane and generate diastolic depolarization (arrows). SR: sarcoplasmic reticulum.

Multiple ionic mechanisms have been reported for the diastolic depolarization causing automatic activity of the pulmonary vein myocardium, including the involvement of intracellular Ca^2+^ (Figure 3). The generation of electrical activity in the guinea pig pulmonary vein myocardium appears to be dependent on intracellular Ca^2+^ concentration. Automatic electrical activity was induced in quiescent preparations by interventions which increase intracellular Ca^2+^ load such as high-frequency pacing [16] or ouabain treatment [8]. In ouabain-treated preparations, the generation of action potential-driven Ca^2+^ transients was preceded by an increase in the intracellular diastolic Ca^2+^ concentration and Ca^2+^ oscillation in the form of Ca^2+^ waves and Ca^2+^ sparks [8]. The inhibition of intracellular Ca^2+^ oscillations by ryanodine completely inhibited the automatic activity. Similar results were reported in the rabbit pulmonary vein, where ryanodine first induced but finally inhibited the electrical activity [48]. In the rat, the pulmonary vein automaticity was inhibited by the inhibitors of phospholipase C and the inositol 1,4,5-triphosphate receptor, which suggests that Ca^2+^ released from the inositol 1,4,5-triphosphate receptor plays an important role [49]. Also, in the angiotensin II-induced automatic activity of the guinea pig pulmonary vein myocardium, intracellular Ca^2+^ sparks and the inositol 1,4,5-triphosphate receptor were involved [50].

Regarding the mechanisms which link intracellular Ca^2+^ to diastolic depolarization and action potential firing, the Na^+^-Ca^2+^ exchanger appears to play a major role (Figure 2) [8]. The diastolic depolarization and action potential firing were inhibited by SEA0400 (1 μM), a highly selective inhibitor of the myocardial Na^+^-Ca^2+^ exchanger [51]; the resting intracellular Ca^2+^ concentration or the Ca^2+^ oscillation was not inhibited. The simplest explanation is that elevated intracellular Ca^2+^ activates the forward-mode Na^+^-Ca^2+^ exchanger, which extrudes Ca^2+^ from the cytoplasm, generating an inward current, and slowly depolarizes the cell membrane [52]. This diastolic depolarization drives the membrane potential to reach the threshold level and generates action potentials. It should be noted that the inhibitory effect of SEA0400 was partial, which suggests the presence of other depolarizing mechanisms in the guinea pig pulmonary vein myocardium. In the case of the rat pulmonary vein, in addition to the Na^+^-Ca^2+^ exchanger current, the involvement of a hyperpolarization-activated chloride current was reported [53]. The channel was further identified to be the CLCN2 channel bound with HSPA8 as an accessory protein [54]. A hyperpolarization-activated inward current was reported in the pulmonary vein cardiomyocytes of the guinea pig, but was small in the rabbit [55]. The species difference in the mechanisms of pulmonary vein automaticity appears to affect their pharmacological properties. The automaticity of the pulmonary vein myocardium in the rabbit was inhibited by ivabradine [56], while that of the rat was inhibited by DIDS [53].

## 5. Involvement of the Persistent Na^+^ Current, Late I_Na_

Another mechanism that can cause diastolic depolarization is the persistent sodium current, late I_Na_ (Figure 3). The voltage-dependent Na^+^ channel current is composed of transient and persistent components. The rapidly activating and rapidly inactivating “transient” component, which is called peak I_Na_, is responsible for the rapid upstroke of the action potential of the working myocardium and the propagation of the action potential through the myocardium [57]. The “persistent” component, which has a very small current amplitude but shows little or no inactivation [58], is referred to as “late I_Na_”, “persistent I_Na_”, or “sustained I_Na_”. Late I_Na_ can be elicited by a long depolarizing step pulse to membrane potentials close to the action potential plateau, or a slowly depolarizing or repolarizing ramp pulse in various voltage ranges, including those corresponding to the diastolic depolarization [59,60,61]. Late I_Na_ is thought to be involved in the ectopic automaticity of the myocardia leading to cardiac arrhythmia [59]. Involvement of late I_Na_ has been suggested in the abnormal automaticity of the atrium and Purkinje fiber [59,61], and in the initiation of atrial fibrillation [62]. The kinetic property of late I_Na_ was reported to be slightly different among canine, rabbit, and guinea pig ventricular cardiomyocytes [63]. Although late I_Na_ continued to flow during the action potential plateau in all three animal species, it was stable during the plateau in the dog and rabbit, while it increased monotonically in the guinea pig. This implies that the effect of drugs acting on the late I_Na_ may be more prominently observed in the guinea pig.

In the guinea pig pulmonary vein myocardium showing spontaneous firing of action potentials, tetrodotoxin, which blocks both the peak I_Na_ and late I_Na_, inhibited the automatic activity through suppression of the diastolic depolarization [64]. In quiescent pulmonary vein preparations, ATX-II, an enhancer of the late I_Na_, induced depolarization of the resting membrane potential followed by induction of automatic activity. GS-458967, which selectively blocked the late I_Na_, suppressed the diastolic depolarization and the firing of action potentials. GS-458967 (1 μM) decreased the diastolic depolarization slope and firing frequency to about half of the initial values. In contrast, pilsicainide (10 μM), which selectively blocks the peak I_Na_ (see below), did not. The existence of late I_Na_ was confirmed in voltage clamp analysis-isolated pulmonary vein cardiomyocytes from the guinea pig; a slowly depolarizing voltage clamp pulse resulted in an inward current in the voltage range corresponding to the diastolic depolarization [64]. The presence of late I_Na_ was also reported in the canine pulmonary vein myocardium [65]. Ranolazine and GS-458967, agents with blocking action on the late I_Na_, inhibited the EAD- and DAD-type electrical activity in the canine pulmonary vein myocardium, although their effects on the diastolic depolarization were not reported [66,67]. NCC-3902 (1 μM), a novel compound which selectively inhibits the late I_Na_ with virtually no effect on other ion channels [68], showed antiarrhythmic effects on a canine atrial fibrillation model [69]. Besides acting as a depolarizing membrane current, the late I_Na_ may also trigger disturbances in intracellular Ca^2+^ homeostasis and hyperactivity of various enzymes, including CaMKII [70,71]. As mentioned above, the forward mode of the Na^+^-Ca^2+^ exchanger contributes to the diastolic depolarization under normal ionic conditions [49,52]. However, under enhanced late I_Na_, the elevated intracellular Na^+^ concentration activates trans-sarcolemmal Ca^2+^ entry through the reverse-mode Na^+^-Ca^2+^ exchanger and may cause intracellular Ca^2+^ overload [71]. Thus, late I_Na_ is probably involved in the diastolic depolarization and automaticity of the pulmonary vein myocardium through its effect as a depolarizing membrane current and as a cause of intracellular Ca^2+^ overload. Thus, agents with inhibitory effects on the late I_Na_ are promising as therapeutic agents for the prevention and treatment of atrial fibrillation.

## 6. Enhancement of Automaticity by Adrenoceptor Stimulation

Regions of the heart, including the pulmonary vein, are densely innervated by the autonomic nerves. The importance of cardiac autonomic innervation in the initiation and maintenance of atrial fibrillation has been demonstrated both experimentally and clinically [72,73]. In the case of the guinea pig pulmonary vein, immunohistochemical detection of noradrenergic nerve fibers [74,75] implied that noradrenaline, released from sympathetic nerve terminals, affects the automaticity. In fact, noradrenaline, as well as tyramine, which releases noradrenaline from sympathetic nerve terminals, induced an automatic activity of the myocardium by enhancing the diastolic depolarization of the action potential [76]. Concerning the adrenoceptor type, both α_1_- and β_1_-adrenoceptors were involved and appeared to act differently. The α_1_-adrenoceptor stimulation triggered the firing of action potentials. The β_1_-adrenoceptor stimulation alone could not trigger action potential firing but increased the firing frequency when applied together with α_1_-adrenoceptor stimulation. At clinically relevant concentrations (10–100 nM), carvedilol, which blocks both α- and β-adrenoceptors, inhibited the noradrenaline-induced activity more effectively than bisoprolol, which selectively blocks β-adrenoceptors. Thus, α_1_- and β_1_-adrenoceptors contribute differentially, and dual stimulation of these two adrenoceptors results in sustained high-frequency firing of the pulmonary vein myocardium.

The mechanisms for the synergistic effect of α- and β-adrenoceptor stimulation were further analyzed in guinea pig pulmonary vein myocardium [76]. α-Adrenoceptor stimulation caused a positive shift in the resting membrane potential and an increase in the slope of diastolic depolarization, which indicates enhancement of time-independent depolarizing ionic currents. Enhancement of the Na^+^-Ca^2+^ exchanger and/or the late I_Na_ are probable candidates for the α-adrenoceptor-mediated mechanisms, but direct evidence awaits further investigation. β-Adrenoceptor stimulation caused a negative shift in the maximal diastolic potential and a shortening of the action potential duration, with only a slight increase in the slope of the diastolic depolarization. β-Adrenoceptor stimulation increases the slow component of delayed rectifier potassium current (I_Ks_) through the activation of protein kinase A [77]. The acceleration of late-phase repolarization and the hyperpolarizing shift of the maximum diastolic potential could be explained by the enhancement of I_Ks_.

The involvement of α- and β-adrenoceptors in the automaticity of the pulmonary vein myocardium has also been reported in rats [12,26,49]. α-Adrenoceptor-stimulation caused a depolarizing shift in the membrane potential and a prolongation of the action potential duration, while β-adrenoceptor-stimulation caused hyperpolarization and a shortening of the action potential duration; simultaneous stimulation of both receptors by noradrenaline resulted in a generation of automatic action potential firing. Selective inhibition of electrical conduction within the pulmonary veins was achieved by α-adrenoceptor stimulation [78]. Although α-adrenoceptor stimulation causes membrane depolarization both in the guinea pig and rat, its effect on automaticity is different. β-Adrenoceptor stimulation can increase the intracellular Ca^2+^ concentration through a cAMP-mediated enhancement of the L-type Ca^2+^ channel, and it may also contribute to automaticity through other mechanisms. The exchange protein activated by cAMP (EPAC) is another downstream effector of β-adrenoceptor stimulation. Knockout of EPAC in mice was shown to decrease the duration of stimulation-evoked atrial fibrillation [79]. Interestingly, the isolated pulmonary vein preparation from EPAC knockout mice showed a lower incidence of spontaneous electrical activity. In human patients, phenylephrine infusion induced atrial fibrillation [80], indicating that both α- and β-adrenoceptors play an important role in the induction of atrial fibrillation, although the involvement of the pulmonary vein was not assessed.

## 7. Effect of Class I Antiarrhythmic Drugs on the Pulmonary Vein Myocardium

Vaughan Williams class I antiarrhythmic drugs, which block the voltage-dependent Na^+^ channel, inhibit the propagation of ectopic excitation through the myocardium [81,82]. Class I antiarrhythmic drugs are used variously depending on their mode of action on the Na^+^ channel, and additional pharmacological and pharmacokinetic properties. Although class I antiarrhythmic drugs are moderately effective against paroxysmal atrial fibrillation, their effectiveness rate is not sufficient and there is room for improvement. This is probably due to the diversity in the pathological conditions among patients and the diversity of the pharmacological properties of individual antiarrhythmic drugs. Class I antiarrhythmic drugs are anticipated to block the conduction of the ectopic action potential originating in the pulmonary vein to the left atria [42]. In the isolated guinea pig pulmonary vein–left atrium connected preparations, the class I antiarrhythmic drug pilsicainide decreased the conduction velocity within the pulmonary vein myocardium as well as that in the left atria [7]. Pilsicainide is a “pure” class Ic antiarrhythmic drug, which blocks the peak I_Na_ with virtually no effect on other major ion channels and transporters [83]. Thus, the inhibitory effects of pilsicainide on conduction could be attributed to the blockade of peak I_Na_, which is a common characteristic of all class I antiarrhythmic drugs. We further examined the effects of several class I antiarrhythmic drugs on the automatic electrical activity of the guinea pig isolated pulmonary vein myocardium [84]. All of the drugs significantly reduced the maximum rate of rise of the action potential, which reflects the blockade of the peak I_Na_ [57]. This confirms that class I antiarrhythmic drugs are indeed expected to inhibit the propagation of ectopic action potentials originating in the pulmonary vein to the left atria similarly to pilsicainide.

**Figure 4:**
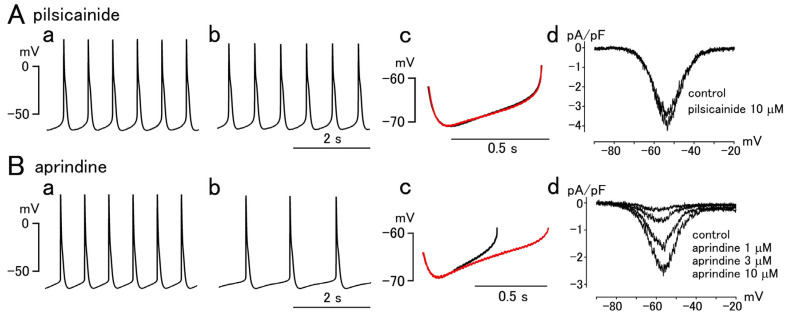
Effect of class I antiarrhythmic drugs pilsicainide and aprindine on the automatic activity of the guinea pig pulmonary vein myocardium and the persistent Na^+^ channel current (late I_Na_) in isolated cardiomyocytes. (**A**) Pilsicainide affected neither the action potential (**a**–**c**) nor the late I_Na_ (**d**). (**B**) Aprindine reduced the firing rate of action potentials (**a**–**c**) and blocked the late I_Na_ (**d**). The action potential recordings were made before (**a**) and 3 min after (**b**) the application of 10 μM of the drugs. Expanded traces of the diastolic depolarization phase before (black) and after (red) drug application were overlaid (**c**). The current–voltage relationship for late I_Na_ under various drug concentrations was displayed (**d**). This figure was adopted from Ref. [84].

The automaticity of the pulmonary vein myocardium, on the other hand, was differentially affected by the class I antiarrhythmic drugs examined (Figure 4) [84]. Cibenzoline, disopyramide, and pilsicainide affected neither the diastolic depolarization slope nor the automatic firing frequency. When the maximum rate of rise of the action potential was decreased by 20% with a concentration of 10 μM of these drugs, neither the firing frequency nor action potential parameters, including the diastolic depolarization slope, was changed. These drugs had no blocking effect on the late I_Na_, elicited by a ramp depolarization in the voltage range including that of the diastolic depolarization. This indicates that the automaticity of the pulmonary vein myocardium cannot be inhibited by blockade of the peak I_Na_ alone. In contrast, aprindine, flecainide, and propafenone significantly decreased the diastolic depolarization slope and significantly reduced the firing frequency. When the maximum rate of rise of the action potential was decreased by 20% with a concentration of 10 μM of these drugs, the firing frequency and the diastolic depolarization slope were reduced by about 20 to 40%, with no changes in other action potential parameters. These drugs showed a blocking effect on the late I_Na_; the concentration–inhibition relationship for the diastolic depolarization slope, firing frequency, and late I_Na_ overlapped. This clear correlation indicates that the blockade of the late I_Na_ is largely responsible for the inhibition of pulmonary vein automaticity by class I antiarrhythmic drugs. Drugs with blocking effects on the late I_Na_ significantly inhibited automaticity while others did not. The effects of conventional class I antiarrhythmic drugs, ajmaline and quinidine, have not been reported. The newly developed late I_Na_-selective blockers ranolazine, GS-458967, and NCC-3902 showed antiarrhythmic effects in vivo [66,67,68,69,85]. These results imply that drugs with blocking effects on the late I_Na_ may be of extra benefit for certain types of atrial fibrillation, in which the automaticity of the pulmonary vein is of particular importance. It was postulated that the focal trigger of pulmonary vein origin is important not only for the initiation but also for the maintenance of atrial fibrillation [86]. The clinical value of late I_Na_ blockers awaits further investigation.

Class I antiarrhythmic drugs may also affect the automaticity of the pulmonary vein myocardium through action on the autonomic nervous system. The pulmonary vein myocardium, as well as the atria, is under the influence of the intracardiac intrinsic neuronal network, which contains both sympathetic and parasympathetic elements [87,88]. Changes in the autonomic nervous tone prior to the onset of atrial fibrillation have been reported [89]. Injection of acetylcholine into a fat pad containing autonomic ganglia at the base of the pulmonary vein caused electrical activity in the neighboring myocardium [90]. The pulmonary vein myocardium shows automatic activity in response to adrenoceptor stimulation, as mentioned above. Thus, class I antiarrhythmic drugs may possibly exert antifibrillatory effects through the blockade of neuronal influence. Cibenzoline and disopyramide, which have anticholinergic activity, were reported to decrease the high-frequency component of heart rate variability [91]. Propafenone, which has β-adrenergic-blocking activity, was reported to decrease the low-frequency component of heart rate variability [92]. Such changes in heart rate variability were not observed with aprindine and pilsicainide, which do not have blocking effects on the receptors [93,94].

Clinical trials with class I antiarrhythmic drugs have shown their effectiveness in the early stage of atrial fibrillation, but not for persistent atrial fibrillation. In patients with acute onset atrial fibrillation, pilsicainide converted 73% of the patients to sinus rhythm [42]. Maintenance of the sinus rhythm for 3 months was observed in 68% and 77% of the patients with pilsicainide and cibenzoline, respectively [95]. On the other hand, in patients with persistent atrial fibrillation, the sinus rhythm was restored in only 22% after 2 weeks [96]. A multichannel blocker, bepridil, achieved conversion to sinus rhythm in 69% of the patients [97]. These results of clinical trials are consistent with the results obtained in animal models mentioned above. It appears that additional mechanisms of action other than the simple blockade of the peak Na^+^ channel current are required for the treatment of persistent atrial fibrillation.

## 8. Negative Inotropic Effect of Class I Antiarrhythmic Drugs

The use of class I antiarrhythmic drugs has not been recommended in patients with compromised cardiac function because of their cardio-suppressive effects [81,82]. While the basic therapeutic effects of class I antiarrhythmic drugs could be explained through their blocking effects on the peak I_Na_ and late I_Na_, the mechanisms for the negative inotropic and chronotropic effects are less understood. The negative inotropic effects of class I antiarrhythmic drugs have been attributed to the Na^+^ blocking effects themselves. The decrease in intracellular Na^+^ concentration caused by Na^+^ channel blockade induces a compensatory intracellular uptake of Na^+^ through the forward mode action of the Na^+^-Ca^2+^ exchanger. The enhanced trans-sarcolemmal efflux of Ca^2+^ results in a reduction in the intracellular Ca^2+^ and contractile force [98]. The negative inotropic effects of several class I antiarrhythmic and related drugs were systematically examined in isolated ventricular papillary muscle preparations to obtain an overall picture of their cardio-suppressive effects [99]. The results revealed a large difference in the negative inotropic effect of drugs. The effect was strong for propafenone and aprindine, intermediate for cibenzoline, flecainide, ranolazine, and disopyramide, and weak with pilsicainide, mexiletine, and GS-458967. The negative inotropic effect was weakened in the presence of SEA0400 and under low extracellular Na^+^ conditions, which suggests that the Na^+^-Ca^2+^ exchanger-mediated mechanism mentioned above is indeed involved in the negative inotropy. However, even under such conditions, the large difference among drugs remained. Further, the negative inotropic effects of drugs were not much affected by the elimination of Na^+^ channel function by elevated extracellular K^+^ concentration. These results clearly indicated that factors other than the Na^+^ channel blockade are largely involved in the negative inotropic action of class I antiarrhythmic drugs.

The voltage-dependent Ca^2+^ channel plays a major role in the regulation of myocardial contraction. The Ca^2+^ that enters the cell through Ca^2+^ channels during the action potential plateau phase triggers Ca^2+^ release from the sarcoplasmic reticulum to form the intracellular Ca^2+^ transient, a process known as the Ca^2+^-induced Ca^2+^ release. Thus, the blockade voltage-dependent Ca^2+^ channel will result in a decrease in the contractile force. In fact, there was a strong correlation between the negative inotropic effects of class I antiarrhythmic drugs and their Ca^2+^ channel-blocking effects. The negative effects of class I antiarrhythmic drugs tended to converge on a single concentration–response curve when the concentration of each drug was expressed as a ratio against its IC_50_ value for Ca^2+^ channel blockade. The contractile force was decreased to about 50% of the initial value at concentrations corresponding to their reported IC_50_ value for the L-type Ca^2+^ channel blockade. Thus, blockade of the L-type Ca^2+^ channel is involved in the negative inotropic action of class I antiarrhythmic drugs, and it is reasonable that the extent of Ca^2+^ channel blockade directly correlates with negative inotropy. This implies that class I antiarrhythmic drugs without Ca^2+^ channel blocking effects are relatively free from cardio-suppressive negative inotropic effects. However, we must keep in mind that the blockade of the peak I_Na_ reduces the conduction velocity and the resulting desynchronization of contraction among ventricular regions may cause a decrease in cardiac output [100].

## 9. Negative Chronotropic Effect of Class I Antiarrhythmic Drugs

The action potential of the sinus node, the orthotopic pacemaker of the heart, has a characteristic waveform different from that of the working myocardium and pulmonary vein myocardium [101]. In cardiomyocytes of the sinus node, which lack I_K1_, the membrane potential oscillates in a depolarized membrane potential range. The maximum diastolic potential of the sinus node is from −55 to −65 mV, at which the voltage-dependent Na^+^ channels are mostly inactivated. Thus, the Na^+^ channel current has only a small contribution to the sinus node action potential, if any. In fact, tetrodotoxin, which blocks both the peak I_Na_ and late I_Na_, has no effect or only a small effect on the sinus node action potential waveform and firing rate [102]. Class I antiarrhythmic drugs, at concentrations overlapping with their therapeutic plasma concentration, decreased the spontaneous beating rate of the guinea pig right atria [103]. There was a difference in the potency among the drugs; cibenzoline, aprindine, flecainide, and propafenone showed stronger effects than disopyramide, mexiletine, pilsicainide, and ranolazine. The IC_50_ values for the negative chronotropy of each drug did not correlate with their IC_50_ values for Na^+^ channel blockade, indicating the involvement of other mechanisms.

In the sinus node, the L-type Ca^2+^ channel current is the most important pacemaking current because the membrane potential range for diastolic depolarization overlaps with the threshold voltage range for the activation of the L-type Ca^2+^ channel. Ca^2+^ antagonists, including class IV antiarrhythmic drugs, block the L-type Ca^2+^ channel and decrease the slope of the diastolic depolarization and the firing rate [101]. The negative chronotropic effect of class I antiarrhythmic drugs could be well explained by their Ca^2+^ channel-blocking effect. When the concentration of each drug was expressed as a ratio against its IC_50_ value for the Ca^2+^ channel blockade, the negative chronotropic effects of all drugs tended to converge on a single concentration-response curve. At concentrations corresponding to their IC_50_ value for L-type Ca^2+^ channel blockade, the firing of the sinus node was decreased to about 80% of the original value. This was the same as the case with nifedipine, which blocks L-type Ca^2+^ channels [104,105]. These results indicated that the negative chronotropic effects of class I antiarrhythmic drugs are mediated by the blockade of the L-type Ca^2+^ channel. The contribution of L-type Ca^2+^ channel blockade to the negative chronotropy appears to be somewhat different from that to inotropy, in which concentrations corresponding to IC_50_ values for the L-type Ca^2+^ channel blockade caused a 50% decrease in the contractile force. The contraction of the myocardium is totally dependent on Ca^2+^ entry through the Ca^2+^ channel for the activation of the CICR mechanism, but in the case of sinus node pacemaking, the L-type Ca^2+^ channel and several other ion channels work additively to form the diastolic depolarization. Dissociation between the negative inotropic and chronotropic effects of class I antiarrhythmic drugs was also observed in an earlier study using canine blood-perfused sinus node and papillary muscle preparations [106].

## 10. Conclusions

The automaticity of the pulmonary vein myocardium could be studied with isolated tissue and cellular preparations from small experimental animals. The diversity in the action potential waveform reflects the difference in the repolarizing potassium channel currents involved. The diastolic depolarization, the trigger of automatic action potentials, is caused by multiple membrane currents, including the Na^+^-Ca^2+^ exchanger current and the late I_Na_. The action potential waveform and automaticity are affected differentially by α- and β-adrenoceptor stimulation. Class I antiarrhythmic drugs block the propagation of ectopic electrical activity of the pulmonary vein myocardium through blockade of the peak I_Na_. Some of the class I antiarrhythmic drugs block the late I_Na_ and inhibit pulmonary vein automaticity. The negative inotropic and chronotropic effects of class I antiarrhythmic drugs could be largely attributed to their blocking effect on the Ca^2+^ channel rather than the Na^+^ channel.
